# Understanding digitalization’s environmental impact: why LCA is essential for informed decision-making

**DOI:** 10.1038/s44168-025-00246-1

**Published:** 2025-04-26

**Authors:** Hannes Bluhm, Daniela Wohlschlager, Johanna Pohl, Severin Beucker, Jan Bieser, Daniel Schien, Kelly Widdicks, Adrian Friday, Gordon Shaw Blair

**Affiliations:** 1 Europa-Universität Flensburg (EUF), Flensburg, Germany; 2 Institute for Ecological Economy Research (IÖW), Berlin, Germany; 3 Technical University of Munich (TUM), TUM Campus Straubing, Straubing, Germany; 4 Intep - IntegralePlanung GmbH, Berlin, Germany; 5 Zurich Knowledge Center for Sustainable Development (ZKSD), Zürich, Switzerland; 6 Borderstep Institute for Innovation and Sustainability, Berlin, Germany; 7 Bern University of Applied Sciences, Institute Public Sector Transformation, Business School, Bern, Switzerland; 8 School of Computer Science, University of Bristol, Bristol, UK; 9 UK Centre for Ecology & Hydrology, Lancaster Environment Centre, Bailrigg, Lancaster UK; 10 School of Computing & Communications, Lancaster University, Bailrigg, Lancaster UK

**Keywords:** Climate-change mitigation, Environmental impact, Technology

## Abstract

This comment critiques Gritsenko et al.‘s dismissal of environmental assessments such as Life Cycle Analysis (LCA) in analyzing digitalization’s environmental impacts. While acknowledging the need for action amidst uncertainty, we argue that LCA yet provides valuable insights into potential impacts, trade-offs, and areas to focus on in a supply chain. Especially in the rapidly evolving digital landscape, LCA helps manage decision-makers’ uncertainty and informs targeted measures for sustainable digital infrastructure deployment and use.

## Introduction

Gritsenko et al.^1^ dispute quantitative approaches to managing the environmental impact of digitalization. The authors argue that with the attempts to quantify and predict the climate effects of digitalization, researchers are “*wasting time and resources trying to gather and estimate quantified information that is not available (or even falling into the false security of invented numbers)*”^1^. This is because the effects are inherently unbounded and unpredictable. Thus, quantification attempts should be avoided: “*we need to move our perception of digital carbo[n] footprint from the realm of ‘unknown’ (so we need to try harder to put a number on it) into the realm of ‘unknowable’ (too complex to model, historical data provide no useful guidance for future outcomes)*”^1^. The authors propose shifting the focus from prediction to mitigation, emphasizing the importance of basing digitalization on renewable energy sources, accounting for dynamic changes in digital development, and managing cost overruns in digitalization projects.

From our viewpoint of championing LCA researchers and practitioners, we appreciate the authors’ conceptual critique of current efforts to assess the environmental impacts of digitalization, especially if this prevents action in the face of uncertainty. We would concur that rather than striving for perfect accuracy of models or models of everything in ultimate detail—which we would regard as a possible ‘discourse of delay’^2^—action *is* needed. However, the conclusions drawn by Gritsenko et al. are flawed, due to misconceptions about the implications of uncertainty for quantitative approaches in general, and LCA in particular resulting in an underestimation of the importance of LCA’s role in strategic decision-making.

Using the more limited framing, yet still important exemplar of LCA^3^, we would point out that *all models are wrong*^4^, *but some are useful*. The process of building LCA models and drawing on expertise to do so, enables comparison, understanding of growth rates, and even identifying the locus of regions of uncertainty all uncover where environmental impacts need addressing or where further questions should be asked. Thus, LCA research on digitalization *directly supports decision-makers* to deal with an uncertain world. Simplifying decision-making to adopting renewables fails to acknowledge the material and environmental implications and dependencies of renewables’ physical infrastructures, and masks essential questions regarding the unchallenged growth of digital infrastructures’ unprecedented demand for energy and materials.

## Diverging results are often not methodological problems but raise important questions

Gritsenko et al. argue that researchers are fixated “*on how to calculate and predict the carbon footprint of digital technologies and initiatives most accurately*”^1^. Arguing that setting sensible system boundaries, including indirect effects and structural changes, and defining the reference system (or baseline) are too challenging and that differing scientific results reveal the inaccuracy of environmental assessments on digitalization.

First, in the case of LCA, the methodological discussion and criticism are as old as the method itself^5^. However, to conclude that LCA is not helpful fails to recognize the method’s aim: analyzing a product system’s potential environmental impacts within defined system boundaries^6^ such that relative comparisons can be made. In comparative analyses, the emphasis is usually not on maximizing accuracy but rather on determining whether the environmental impacts of the two systems (e.g., video conferencing versus physical travel) are comparable. LCA’s ability to assess a product system across different environmental impact categories helps avoid unintended burden shift^7^, e.g., from less climate change to more resource depletion, as with the proposed mitigation strategy.

Second, analyzing the indirect impacts, structural, and future effects of product systems in LCA are vivid research fields, namely Consequential and Prospective LCA^5^. In addition, scenarios are not assessments intended to predict the future as accurately as possible. Börjeson et al.^8^ distinguish predictive, explorative, and normative scenarios, which address different goals and can be informed or integrated with LCA. Thus, LCA is not the source of uncertainty but rather a method to address uncertainty about developments by assessing different pathways, finding measures for improvement and intervention, and, eventually, mitigating critical developments^9^.

Third, Gritsenko et al. argue that scenario-based estimates of greenhouse gas emissions carry significant risks, often produce conflicting results which is inter alia “*reflected in how previous studies have yielded inconsistent results on the direct climate effects of digitalization*”^1^, and thus they critique the overall usefulness of such analyses. However, diverging results of LCAs are not necessarily a sign of contradiction: they can also be rooted in diverging goal and scope definitions which require different system boundaries, methodological choices, and assumptions, all of which are important to surface. Some consensus has emerged regarding the ICT sector’s carbon footprint, confirming that the sector currently accounts for approximately 1.5–4% of global greenhouse gas emissions^10,11^. Even though such results are still challenged, a critical reflection on the methods applied, the data quality and assumptions, and their influences on the results is good scientific practice that improves assessment results and informs mitigation measures.

## LCA methodology offers a broader analytical scope beyond the mere determination of (digital) carbon footprints

We argue that aiming to “*predict digital carbon footprint accurately*”^1^ is only one of many possible goals of LCA. Among other purposes, it is also about understanding the environmental impacts of a digital system in the present and the future, in assumed static and dynamic environments, as well as in comparison to other systems, identifying hotspots of environmental impacts and finding measures for improvement^5^. For example, assessments of smart energy use cases comparing direct vs. bidirectional charging of electric vehicles^12^ or analyses on smart homes^13^ have revealed valuable insights on net impacts, critical life-cycle steps, potential trade-offs between environmental categories, and possible alternatives. The research question here is often not about whether to digitalize but how different digital use cases compare and how environmental impacts can be minimized. As another example, various LCAs of digital end-user devices have shown that the production causes significantly higher climate effects than the use phase^14^, directing mitigation efforts from use phase toward the supply chain and maximizing product life.

The direct implications of digitalization are not completely “*unknowable*” since the bill of materials and energy use are comparably robust data points^15^. Indirect effects are much harder to assess, requiring further inventory data and calls for interdisciplinary approaches, e.g., by involving methods from behavioral sciences or economics^16^. However, much proof of combining methods in the case of digitalization that led to insightful and actionable results exists (e.g., ref. 13).

## Discontinuing LCA research on digitalization and solely focusing on renewable energy supply ignores decision-makers’ needs and implies an unquestioned burden shift

Gritsenko et al. explain that *“[d]ecision-making under risk requires accurate data for calculating probabilities and optimizing the current course of action. Knightian or radical uncertainty [as in the case of digitalization] requires decision-making beyond the numbers because reliable data are unavailable*”^1^. Therefore, they suggest that “*if digitalization were entirely based on renewables, […] developing tools to quantify and estimate these links would become less relevant*”^1^. Do we echo a need to act without modeling everything? Certainly. However, such heuristic reasoning beyond models is highly risky because they are based on limited knowledge and prone to biases and preconceptions^17^: technologies for harvesting renewable energy sources are not impact-free throughout their life cycle nor homogeneous in their performance, including trade-offs between different life cycle impact categories^18^. Furthermore, Gritsenko et al. do not mention that their preconception that renewable energies are low in carbon footprint (which they claim mitigates digitalization’s direct and indirect effects) is likely based on predictions and assessments that they in turn reject for the evaluation of digitalization.

Better, we argue, reduce demand, consider growth limits, and question innovations’ impact as early as possible with *necessary* models. Only focusing on harvesting renewables inadvertently shifts the burden from climate change to other environmental rebounds, such as (scarce) raw materials, land use, and sovereignty of power systems. LCAs are *particularly useful* in revealing such trade-offs – identifying levers to reduce direct and indirect negative impacts and enable potential positive effects of digitalization. For example, assessments have shown that both direct and indirect effects of digitalization can increase mineral raw material depletion and impacts that go with it (e.g., additional human and ecosystem toxicity) while enabling greenhouse gas mitigation^19,20^.

Finally, Gritsenko et al. recognize the continuous dynamic change in the technological landscape and argue that it makes assessments such as those based on LCAs less relevant. While acknowledging that this makes it challenging to quantify precise values, this dynamic change and the resulting uncertainty from innovation and growth are *actually what drive the demand for LCAs* from decision-makers in industry, politics, and civil society. In fact, we notice that decision-makers demand LCAs and meaningful insights from independent scientific institutions precisely because they are experienced in weighing alternatives beyond simplified heuristics.

Future sustainability assessments of digitalization’s environmental impacts, such as LCA, should embrace the inherent (non-quantified) uncertainty that comes with it. In practice, this could mean combining quantitative uncertain assessments with additional qualitative risks and uncertainty evaluation drawing on expert insights^21^.

## Data Availability

No datasets were generated or analysed during the current study.
